# Recommendations for the analysis of individually randomised controlled trials with clustering in one arm – a case of continuous outcomes

**DOI:** 10.1186/s12874-016-0249-5

**Published:** 2016-11-29

**Authors:** Laura Flight, Annabel Allison, Munyaradzi Dimairo, Ellen Lee, Laura Mandefield, Stephen J. Walters

**Affiliations:** 1 ScHARR, University of Sheffield, 30 Regent Street, Sheffield, S1 4DA UK; 2 MRC Biostatistics Unit, Cambridge Institute of Public Health, Forvie Site, Robinson Way, Cambridge Biomedical Campus, Cambridge, CB2 0SR UK

**Keywords:** Clustering, Randomised controlled trial, Statistical models, Therapist effects, Individually clustered randomised controlled trials

## Abstract

**Background:**

In an individually randomised controlled trial where the treatment is delivered by a health professional it seems likely that the effectiveness of the treatment, independent of any treatment effect, could depend on the skill, training or even enthusiasm of the health professional delivering it. This may then lead to a potential clustering of the outcomes for patients treated by the same health professional, but similar clustering may not occur in the control arm. Using four case studies, we aim to provide practical guidance and recommendations for the analysis of trials with some element of clustering in one arm.

**Methods:**

Five approaches to the analysis of outcomes from an individually randomised controlled trial with clustering in one arm are identified in the literature. Some of these methods are applied to four case studies of completed randomised controlled trials with clustering in one arm with sample sizes ranging from 56 to 539. Results are obtained using the statistical packages R and Stata and summarised using a forest plot.

**Results:**

The intra-cluster correlation coefficient (ICC) for each of the case studies was small (<0.05) indicating little dependence on the outcomes related to cluster allocations. All models fitted produced similar results, including the simplest approach of ignoring clustering for the case studies considered.

**Conclusions:**

A partially clustered approach, modelling the clustering in just one arm, most accurately represents the trial design and provides valid results. Modelling homogeneous variances between the clustered and unclustered arm is adequate in scenarios similar to the case studies considered. We recommend treating each participant in the unclustered arm as a single cluster. This approach is simple to implement in R and Stata and is recommended for the analysis of trials with clustering in one arm only. However, the case studies considered had small ICC values, limiting the generalisability of these results.

**Electronic supplementary material:**

The online version of this article (doi:10.1186/s12874-016-0249-5) contains supplementary material, which is available to authorized users.

## Background

Randomised controlled trials (RCTs) are commonly used to evaluate the efficacy of healthcare treatments where patients are randomised to receive care from the same source; for example a health professional such as a nurse, therapist, general practitioner (GP) or surgeon. There are two main types of RCTs: group/cluster randomised controlled trials (cRCTs) and individually randomised controlled trials (iRCTs). Cluster RCTs randomise groups or clusters (of individuals) to the treatment arms; for example GP practices, schools or communities whilst iRCTs randomise individual patients [1, 2]. In a cRCT, for example, where patients in each treatment arm receive one of two group based interventions, we might expect patients in the same group to experience similar outcomes purely as a result of their group allocation. It is important to try and account for this cluster or group effect when designing and analysing the data.

RCTs where individuals are randomised are not immune to this clustering effect either. In an iRCT where the treatment is delivered by a health professional it seems likely that the effectiveness of the treatment, independent of any treatment effect, could depend on the skill, training or even enthusiasm of the health professional delivering it. This may then lead to a potential clustering of the outcomes for patients treated by the same health professional or who received treatment as a group. Alternatively a single therapist may deliver an intervention to a sample of patients on an individual basis while another therapist delivers the intervention to a different sample of patients. We might expect there to be clustering in the patients who received treatment from the same therapist. In both cRCTs and iRCTs with clustering we can measure the extent to which outcomes within the same cluster may depend on each other using the intra-cluster correlation coefficient (ICC) [2].

If the outcomes are clustered then the conventional statistical methods for analysing RCT outcome data, such as an independent two sample *t*-test to compare the mean outcomes between the treatment and control groups, may not be appropriate as the methods assume the observed outcomes on different patients are independent [3]. When there is clustering there is a lack of independence among the outcomes. When using conventional statistical methods this may lead to underestimation of the standard error for the treatment effect estimate, narrower confidence limits and hence larger values for the test-statistic (the ratio of the treatment estimate to its standard error) and smaller *P*-values. The extent to which the results are affected depends on the average cluster size in the trial and the magnitude of the ICC [4]. For example a high ICC (≥0.05) may not greatly impact the results if the average size of the clusters is small and a low ICC (<0.05) may have a large impact on the results if the average cluster size is large. If we do not use appropriate methods to allow for this we can underestimate the standard error and over-estimate the significance of results. Furthermore, there is a reduction in the evaluable sample size and so the power of the study to detect a treatment effect decreases.

Using the nomenclature of Baldwin [5], the clustering that arises in iRCTs can be split into two categories, fully clustered and partially clustered. A fully clustered trial is one with elements of clustering that span both arms of the trial. An example of a fully clustered trial is one comparing homeopathic remedy with placebo for the treatment of chronic fatigue syndrome [6]. Patients were assigned to a homeopath and then within each homeopath the patients were randomly assigned to either the treatment or control. As patients on both treatments saw the same homeopath there is clustering by homeopath in each arm of trial.

**Fig. 1:**
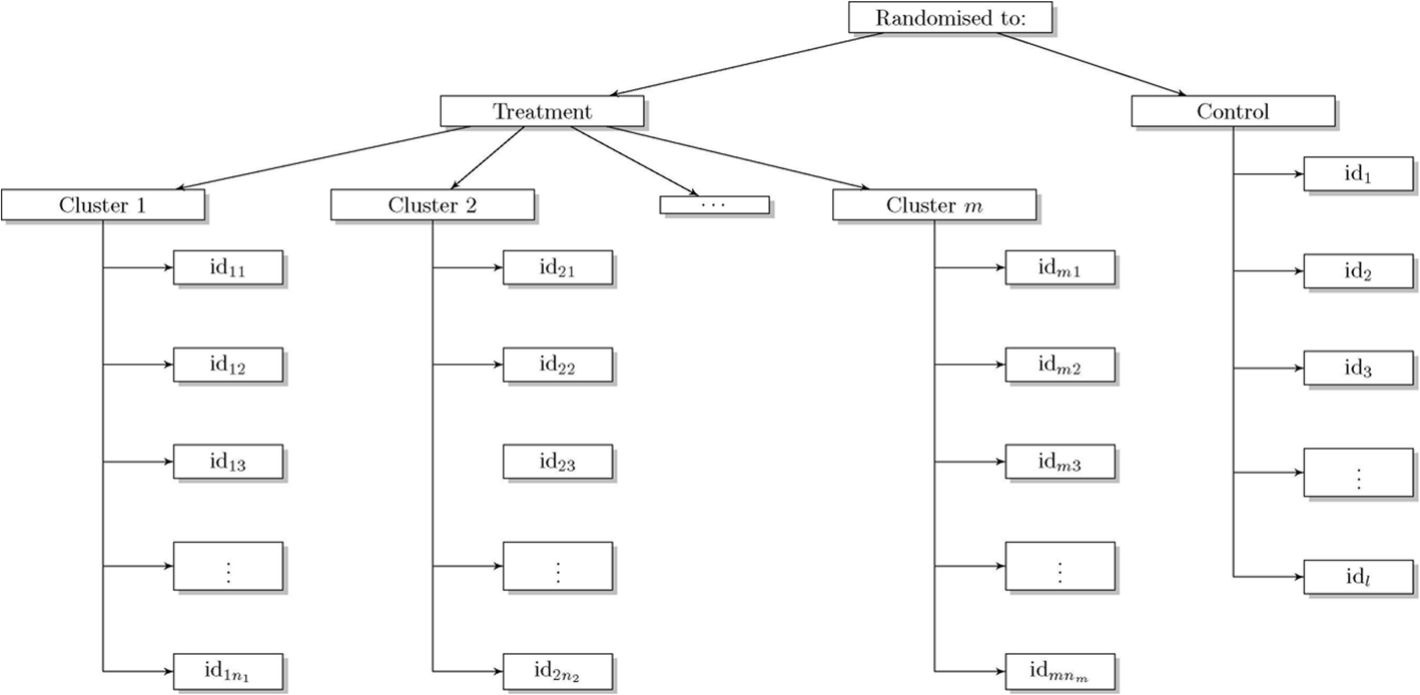
Schematic of a trial with clustering in only one arm (the treatment arm) where *n*_1_,…,*n*_*m*_ is the number of patients in the *m* treatment clusters (clusters are not necessarily of equal size but this is often fixed in advance) and *l* is the number of subjects in the control arm

Partially clustered designs describes a trial where clustering occurs in just one of the arms of the trial. An example of a partially clustered design is a trial comparing acupuncture with usual care for the treatment of persistent non-specific low back pain [7]. Patients in the treatment arm were treated by one of the trial acupuncturists. Clustering occurs in one arm of the trial only, where a health professional-given treatment is being compared with usual care. There is clustering by heath professional in the treatment arm but no equivalent clustering in the control arm (Fig. 1).

This paper reviews and describes the statistical methods for analysing outcomes from an iRCT with some element of clustering in one arm. We focus on trials with continuous outcomes and assume the clustering occurs in the treatment arm only. We explore the performance of all the models including naïve approaches that were implemented in our case studies prior to the development of more sophisticated methods. We provide practical guidance and recommendations for the analysis of iRCTs with some element of clustering in only one arm.

## Methods

### Literature search

A comprehensive literature search was used to identify published work on clustering in iRCTs. A search of the database MEDLINE was conducted on 1^*st*^ August 2014. The following search criteria were implemented:



Two statisticians (LF and EL) hand searched the articles independently based on titles, abstracts and where necessary the full article, to identify relevant results. Relevant articles contained details of RCTs with clustering in one arm or methods used to analyse such trials. In addition to the database search, papers known by the authors to be relevant were included. Researchers known to be working in this area were contacted to identify unpublished or ongoing work.

A consensus decision was then made between LF and EL as to relevant articles. This list was then reviewed and summarised, identifying the most relevant articles for this project - those describing methodology for handling clustering in one arm of iRCTs.

#### Literature search results

The MEDLINE search identified 353 articles. After the initial hand searching exercise 22 (19 from the MEDLINE search and three from other sources) were shortlisted and 17 were included in the list of relevant articles. These articles included methodological and application papers providing methods for the analysis of trials containing clustering in one arm and are referenced throughout.

## Models

The following models were selected based on the findings of the literature search. The general notation is as follows; *y* denotes the continuous outcome, *i* is the patient indicator, *j* is the cluster indicator, *t* is the treatment indicator variable, *β*_0_ is the intercept and *θ* is treatment effect.

### Simple regression

The most straightforward and naïve option for the analysis of trials with clustering in one arm is to ignore clustering and use a simple linear regression model. This model assumes observations within the same treatment arm and cluster are independent. Here *y*_*i*_ is a continuous outcome for patient *i*, *t*_*i*_ is the treatment indicator variable (*t*=0 for control and *t*=1 for the treatment arm) for patient *i*, *θ* is the treatment effect, *ε*_*i*_ are Normally distributed errors with mean zero and residual variance $\sigma ^{2}_{\epsilon }$. This represents the patient level variation.

1 $$\begin{array}{*{20}l}  y_{i}&=\beta_{0}+\theta t_{i}+\epsilon_{i}, \end{array} $$

2 $$\begin{array}{*{20}l} \epsilon_{i} &\sim N\left(0,\sigma_{\epsilon}^{2}\right). \end{array} $$

Although this model is simple to implement and common in practice it may give incorrect results as the independence assumption of the linear regression model is violated [8]; standard errors of parameter estimates and the *p*-value are likely to be smaller than they should be [2]. This will depend on the level of clustering as measured by the ICC and the average cluster size.

### Imposing clustering in the control arm

Rather than ignoring the clustering in the trial we can account for it in the model used for analysis. As there is clustering in just one arm of the trial, one option is to impose clusters on the control arm that in reality do not exist. This will allow the implementation of methods used in the analysis of cRCTs with clustering in both arms. There are different options for imposing clusters (*j*) in the control arm. Table 1 gives three different options where *l* is the number of participants and *k* is the number of arbitrary clusters in the control arm. The first option treats the control arm as a large artificial cluster of size one [9]; the second option treats each individual within the control arm as a cluster of size one with *j*=*l* clusters in the control arm [5, 8, 10]. Both approaches may cause problems when estimating the ICC as, in theory, it is not possible to estimate between cluster variability in the control arm (Option 1, Table 1) and within cluster variability in the control arm (Option 2, Table 1). However, in practice, the exclusive person-to-person variability in the control arm is artificially partitioned into the between and within cluster components that occur with the treatment arm [5]. The third option overcomes the issue of estimating the ICC. We create artificial-random clusters in the control arm as in Option 3 (Table 1) [9]. Consideration may be given to the number of arbitrary clusters (*k*) to minimise bias in the estimation of treatment effect. There is paucity of literature guiding the optimum choice of the artificial-cluster sizes, hence for pragmatic and simplicity reasons *k* could be chosen to ensure cluster size is roughly equal across treatment arms.

**Table 1:** Different options for imposing clustering of controls

Option	Control	Treatment
1	*j*=0	*j*=1,…,*J*
2	*j*=1,…,*l*	*j*=*l*+1,…,*J*
3	*j*=1,…,*k*	*j*=*k*+1,…,*J*

#### Cluster as a fixed effect

It is possible to account for clustering by including cluster as a fixed covariate [5]; treating cluster coefficients as nuisance parameters. In Eq. 3 *y*_*ij*_ is the outcome for patient *i* in cluster *j*, *β*_*j*_ is the cluster effect, *c*_*j*_ is the cluster indicator.

3 $$\begin{array}{*{20}l}  y_{ij}&=\beta_{0}+\theta t_{ij}+\sum\limits_{j=1}^{J}\beta_{j}c_{j}+\epsilon_{i}, \end{array} $$

4 $$\begin{array}{*{20}l} \epsilon_{i} &\sim N\left(0,\sigma_{\epsilon}^{2}\right). \end{array} $$

While the fixed effect model may appear simple, fitting the fixed effects model is not straightforward as the model will be over-fitted; not all parameters in the model can be estimated since within each cluster each participant receives only the intervention or the control [11]. Consequently, by setting one cluster to be the reference category the between cluster treatment effect cannot be easily estimated. There is no cross classification for treatment arm. While options are available for fitting this model, we do not advocate this approach [5]. The fixed effects model does not truly reflect the study design. Therefore we will not consider the model further in this paper.

#### Cluster as a random effect

Using a random effects model mitigates some of the limitations of the fixed effects model. The inclusion of a random cluster effect adds just one parameter for estimation in the model, rather than *J*−1 parameters as in Eq. 3 [12]. This increases the degrees of freedom and allows exploration of the different sources of variability; between and within cluster. In this model we fit a random intercept for each cluster (*u*_*j*_) and assume it is Normally distributed with zero mean and cluster effect variance $(\sigma _{u}^{2})$. Here, *ε*_*ij*_ is the patient level variation for the *i* ^*th*^ patient in the *j* ^*th*^ cluster.

5 $$\begin{array}{*{20}l}  y_{ij}&=\beta_{0}+\theta t_{ij}+u_{j}+\epsilon_{ij}, \end{array} $$

6 $$\begin{array}{*{20}l} u_{j} &\sim N\left(0,{\sigma_{u}^{2}}\right), \end{array} $$

7 $$\begin{array}{*{20}l} \epsilon_{ij} &\sim N\left(0, \sigma_{\epsilon}^{2}\right). \end{array} $$

Again, as with Eq. 3 the imposed clustering of the control arm must be selected (Table 1, Options 1 to 3).

### Modelling clustering in one arm

Imposing clustering in the control arm is theoretically not an ideal solution [5]. Alternatively we can consider models that do not force any clustering on the ‘unclustered’ control arm, instead we model just the clustering in the treatment arm. Subjects in the control arm are assumed to be independent [5]. As such the ICC is allowed to vary between the intervention and the control arm. Here the ICC in the control arm is modelled to be zero and in the intervention arm is modelled using Eqs. 19 and 20 given later. This partially clustered approach [8, 10, 13], more accurately reflects the nature of the clustering in the trial design [5], so is seemingly preferable to the forcing clustering methods.

#### Partially clustered model

In this model we confine the random effect to the treatment arm only, and hence do not need to configure artificial-clusters as in Table 1.

8 $$\begin{array}{*{20}l}  y_{ij}&=\beta_{0}+\theta t_{ij}+t_{ij}u_{j}+\epsilon_{ij}, \end{array} $$

9 $$\begin{array}{*{20}l} u_{j} &\sim N\left(0,{\sigma_{u}^{2}}\right), \end{array} $$

10 $$\begin{array}{*{20}l} \epsilon_{ij} &\sim N\left(0, \sigma_{\epsilon}^{2}\right). \end{array} $$

We define a random slope model, however when writing out the models for the two levels of *t*_*ij*_ we can see this essentially amounts to a random intercept for each cluster in the treatment arm only (Eq. 11) and one intercept for the unclustered control arm (Eq. 12).

11 $$\begin{array}{*{20}l}  \text{For the treatment arm } (t_{ij}&=1):\\ &y_{ij}=\beta_{0}+\theta+u_{j}+\epsilon_{ij}. \end{array} $$

12 $$\begin{array}{*{20}l} \text{For the control arm } (t_{ij}&=0):\\ &y_{ij}=\beta_{0}+\epsilon_{ij}. \end{array} $$

#### Heteroskedastic individual level errors

In the partially clustered model (Eq. 8) the individual level errors *ε*_*ij*_ have the same variance in the control and the treatment arm - hence the model is homoscedastic. An extension of this allows for different individual level errors in the two treatment arms. In a trial with therapists delivering an intervention in the treatment arm and no intervention in the control arm we might expect participants in the treatment arm to vary in a different way to those participants in the control arm. The outcome might be more homogeneous in participants in the treatment arm as between therapist variation is small due to adherence strict protocols for treatment implementation. It is possible to extend the partially clustered approach to allow for heteroskedastic errors between the treatment arms [5, 8, 13]. The intervention arm varies differently to the control arm. Here

13 $$\begin{array}{*{20}l}  y_{ij}&=\beta_{0}+\theta t_{ij}+t_{ij}u_{j}+(1-t_{ij})r_{ij}+t_{ij}\epsilon_{ij}, \end{array} $$

14 $$\begin{array}{*{20}l} r_{ij} &\sim N\left(0,{\sigma_{r}^{2}}\right), \end{array} $$

15 $$\begin{array}{*{20}l} u_{j} &\sim N\left(0,{\sigma_{u}^{2}}\right), \end{array} $$

16 $$\begin{array}{*{20}l} \epsilon_{ij} &\sim N\left(0, \sigma_{\epsilon}^{2}\right). \end{array} $$

For the treatment arm the cluster level error is *u*_*j*_ and the individual level error is *ε*_*ij*_ (Eq. 17) and in the control arm the individual level error is *r*_*ij*_ (Eq. 18).

17 $$\begin{array}{*{20}l}  \text{For the treatment arm} \left(t_{ij}=1\right)&:\\ y_{ij}=&\beta_{0}+\theta+u_{j}+\epsilon_{ij}. \end{array} $$

18 $$\begin{array}{*{20}l} \text{For the control arm} \left(t_{ij}=0\right)&:\\ y_{ij}=&\beta_{0}+r_{ij}. \end{array} $$

This model can reveal whether individuals become more homogeneous in their attitudes and behaviours as a function of treatment arm membership [8].

**Fig. 2:**
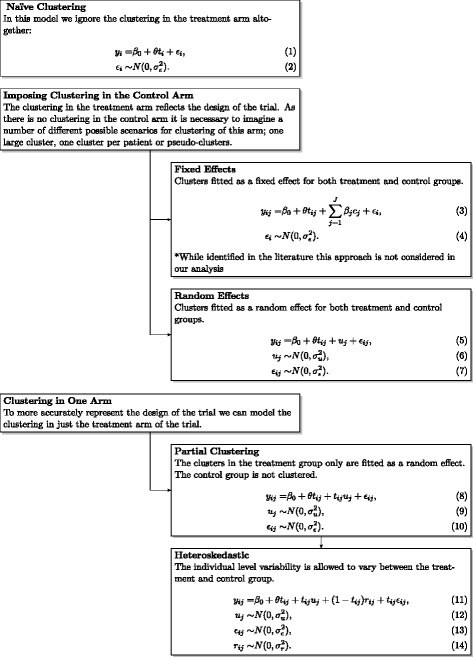
Summary of models for the analysis of iRCTs with clustering in one arm only *y* denotes the continuous outcome, *i* is the patient indicator, *j* is the cluster indicator, *t* is the treatment indicator variable (*t*=1 for the treatment arm and *t*=0 for the control arm), *θ* is treatment effect, *ε*, *u* and *r* are error terms

A summary of the models that can be used in the analysis of iRCT with clustering in one arm is given in Fig. 2.

For the random effects, partially clustered and heteroskedastic models it is possible to estimate the ICC to measure the overall level of clustering in the trial across both arms [2]. For the random effects and partially clustered models we use

19 $$\begin{array}{*{20}l}  ICC=\frac{{\sigma_{u}^{2}}}{{\sigma_{u}^{2}}+\sigma_{\epsilon}^{2}}. \end{array} $$

The heteroskedastic model requires an additional term in the denominator as we have now allowed the residual variance to differ between the treatment and control arms. This formula was adapted from the work of Roberts (2010) [14] on nested therapist designs

20 $$\begin{array}{*{20}l}  ICC=\frac{{\sigma_{u}^{2}}}{{\sigma_{u}^{2}}+\sigma_{\epsilon}^{2}+{\sigma^{2}_{r}}}. \end{array} $$

### Case studies

We compared 10 models using four example case studies from iRCTs with clustering in one arm: specialist clinics for the treatment of venous leg ulcers [15], acupuncture for low back pain [7], cost-effectiveness of community postnatal support workers (CPSW) [16], and Putting Life in Years (PLINY) [17]. These studies were selected from our portfolio of studies as trial statisticians that had clustering in one arm only. The trials are summarised in Table 2.

**Table 2:** Summary of the case studies

Trial	Leg Ulcers	Acupuncture	CPSW	PLINY
Objective	Establish clinical effectiveness of specialist community leg ulcer clinics versus usual care provided by district nurses [15]	Determine whether a short course of traditional acupuncture improves longer-term outcomes for patients with persistent nonspecific low back pain [7]	Establish the relative cost-effectiveness of postnatal support in the community in addition to the usual care provided by community midwives [16]	Evaluate the effectiveness and cost-effectiveness of telephone befriending for the maintenance of health related quality of life (HRQoL) in older people [17]
Cluster	8 specialist clinics	7 acupuncturists	7 CPSW	5 volunteer facilitators
Outcome of Interest	^a^Number of ulcer free weeks during 12 months follow-up	SF-36 pain dimension measured at 12 months follow-up [28]	SF-36 general health perception domain measured at 6 weeks [28]	SF-36 mental health dimension score measured at 6 months follow-up [28]
Target Difference		10 points	5 points	8 points
Original Analysis	–	Robust standard errors	No adjustment	Generalised linear model with robust standard errors with participants in the control arm treated as individual clusters of size one

^a^This was not the primary outcome in the main study

### Main analysis

The clustering structure of each case study was first summarised by the number of clusters in the treatment arm and the mean, median, minimum (min), maximum (max) and inter-quartile range (IQR) of the cluster size. All analyses used complete cases for simplicity; patients with data missing for the primary outcome were removed. Box plots aided visualisation of the spread of data within and between each cluster for each case study. Patients with missing cluster allocation in the treatment arm were grouped as one cluster in both the summary table and the box plots.

#### Model fitting

To explore the practical aspects of the models proposed for analysing an iRCT with clustering in one arm we used two statistical packages – Stata and R. The results presented here are taken from the analysis in R [18] as Roberts has comprehensively presented results using Stata [14]. Scripts for both packages are provided (see Additional file 1).

All models were fitted using a restricted maximum likelihood procedure (REML) and the following specifications of the clustering in the control arm were used:

1. Treating controls as clusters of size one,
2. Treating controls as one large cluster,
3. Creating artificial-clusters.

Although in theory we do not model the clustering in the control arm for both the partially clustered and heteroskedastic models, for the sake of running a model in R or Stata it is necessary to impose clustering. All three approaches are explored. The artificial-clusters in the control arm were created by randomly assigning control patients to a cluster based on the average cluster size in the intervention arm.

When analysing clustered data with small to medium number of clusters, a correction to the degrees of freedom is recommended to protect against inflation of type I error [19]. A number of methods which include Satterthwaite [20] and Kenward-Roger [21] approximations have been proposed to correct degrees of freedom. The debate about which procedure to adopt and under what circumstances is beyond the scope of this paper. In this study, the results were however similar regardless of whether a correction to the degrees of freedoms was made or not. In this regard, the results are presented using REML approximation without any correction to the degrees of freedom. However, Stata’s mixed command allows the correction of degrees of freedom using a number of methods including Satterthwaite and Kenward-Roger approximations [22].

Using R, the model ignoring clustering was fitted using the lm() command [18] in the stats package. The lme4 package was used to fit the random effects and the partially clustered model however it was not possible to use the same package for the heteroskedastic model as this package does not allow heteroskedastic errors [23]. Instead the nlme and lme() function were used [24]. Bespoke functions were written in R to calculate the ICC for the appropriate models as per Eqs. 19 and 20.

The lme4 package does not produce *p*-values for model estimates and so does not need an estimate for degrees of freedom. This omission is due to the authors not supporting current approaches for doing so [23]. The nlme package uses approximations to the distributions of the maximum likelihood estimates to produce *p*-values. This method requires an estimate of degrees of freedom which is outlined in detail by Pinheiro and Bates [25].

The results from each model were compared visually using a forest plot and summarised in a table.

## Results

### Summary of case studies

**Fig. 3:**
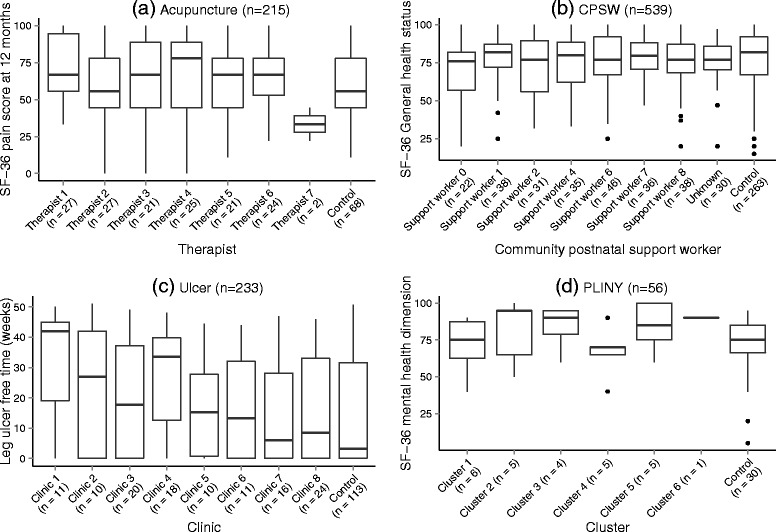
Box plot of the case studies. Patients with missing outcome data have been removed

Table 3 provides a summary of the four case studies considered. The CPSW case study has the largest amount of missing outcome data (13.5%), all patients with no outcome data were removed from the model fitting. Figure 3 shows there is slight variation in the median general health perception domain of the SF-36 with clear differences in the spread of the data depending on the support worker. This indicates small potential for clustering of outcomes in the treatment arm.

**Table 3:** Summary of the clustering in the case studies where IQR is the inter-quartile range. The summary of the cluster sizes is based on patients with a valid primary endpoint (number analysed)

	Total randomised	Missing	No. analysed (control)	No. clusters	Mean	Median (IQR)	(Min, max)
CPSW^a^	623	84	539 (263)	8	34.5	35.5 (7.25)	(22, 46)
Acupuncture	239	24	215 (68)	7	21.0	24.00 (5.00)	(2, 27)
Ulcer	233	0	233(113)	8	15.0	13.50 (7.75)	(10, 24)
PLINY^a^	70	14	56 (30)	6	4.3	5.00 (0.75)	(1, 6)

^a^Trial grouped participants with no cluster allocation in the treatment arm into a single cluster

The Acupuncture study had an average cluster size of 21 in the intervention arm and 80 patients in the control arm. As with each of the case studies the controls were randomly assigned to artificial-clusters. Here four clusters of size 20 were used. Therapist 7 saw two patients, much fewer than the other therapists. In Fig. 3 the median pain score at 12 months varies slightly between the therapists (not accounting for Therapist 7) and there is little variability when compared to the control arm. Again there is potential for clustering of outcomes in the treatment arm.

The outcome of interest in the Ulcer case study is recorded for all patients in this case study. Figure 3 shows great variability in the median leg ulcer free weeks between clinics and in comparison to the controls, indicating potential clustering of the outcome in the treatment arm.

The PLINY case study was a pliot trial and as such had an evaluable sample size of 56. There were five patients in the treatment arm with no cluster allocation. As the other clusters in the treatment arm were of size six, we grouped the five patients without cluster allocation into their own cluster. In Fig. 3, four of these patients had missing outcome data. All clusters contain only a few patients (a maximum of 6), a reflection of the small sample size for this study. The small number of patients in each cluster makes it difficult to assess any variability between facilitators in Fig. 3 and the control arm. There is some suggestion of variability in the median score in the mental health domain of the SF-36 indicating potential for clustering in the outcome dependent on the facilitator.

### Models

The results from fitting the models to the CPSW case study are given in Table 4. The estimate of the treatment difference and its standard error for the model ignoring clustering and the random effects, partially clustered and heteroskedastic models are all similar, including for the various imposed clustering options in the random effects model. The residual variance is comparable for all these models and the random variation (where applicable) is small (<0.0001).

**Table 4:** Summary of results for the CPSW case study (*n*=539)

Model	Treatment estimate	Standard error	Residual variance	Random variance	Control variance	ICC
Ignore clustering	-1.62	1.60	343.31			
Random effects						
Individual clusters of size 1	-1.62	1.60	343.31	<0.0001		<0.0001^a^
One large cluster	-1.62	1.60	343.31	<0.0001		<0.0001^a^
Artificial-clusters	-1.62	1.60	343.31	<0.0001		<0.0001^a^
Partially clustered						
Individual clusters of size 1	-1.62	1.60	343.31	<0.0001		<0.0001^b^
One large cluster	-1.62	1.60	343.31	<0.0001		<0.0001^b^
Artificial-clusters	-1.62	1.60	343.31	<0.0001		<0.0001^b^
Heteroskedastic						
Individual clusters of size 1	-1.62	1.60	339.42	<0.0001	347.38	<0.0001^b^
One large cluster	-1.62	1.60	339.42	<0.0001	347.39	<0.0001^b^
Artificial-clusters	-1.62	1.60	339.42	<0.0001	347.39	<0.0001^b^

^a^ICC across both arms of the trial

^b^ICC in the intervention arm only

**Fig. 4:**
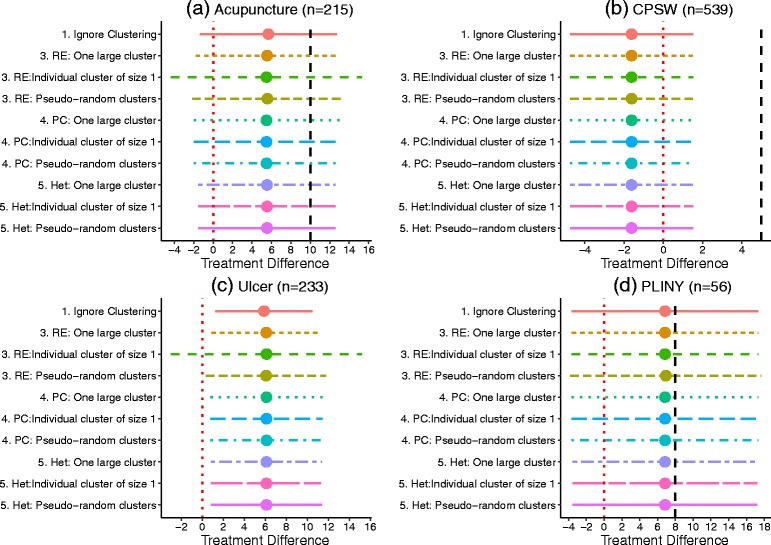
Forest plot of models fitted using R for each of the case studies where RE is random effects, PC is partial clustering, Het. is heteroskedastic model. The vertical, *black dashed line* represents the target treatment difference. We are not using the primary outcome from the Ulcer case study and so this line is not marked. The vertical, *red dotted line* marks a zero treatment difference

For the random effects model in the remaining case studies there is some dependence on how the clustering in the control arm is specified. For example, in the Acupuncture case study the estimate of the treatment difference ranges from 5.49 to 5.59 and its standard error from 3.75 to 5.02 (Table 5). This is evident in the forest plot in Fig. 4. A similar result is found for the Ulcer case study (Table 6) with the standard error greatest when the controls are treated as one large cluster. The choice of imposed clustering method also affects the residual error and the random error. For the PLINY case study (Table 7) the standard error is largest when the controls are treated as artificial-clusters. This suggests the specification of clustering in the control arm can influence the results when using a random effects model. A possible explanation is the small number of patients per cluster. In the case studies the within cluster variance is estimated with large uncertainty. As expected, the partially clustered and heteroskedastic models appear not to depend on the specification of controls giving identical results regardless of the approach adopted.

**Table 5:** Summary of results for the Acupuncture case study (*n*=215)

Model	Treatment estimate	Standard error	Residual variance	Random variance	Control variance	ICC
Ignore clustering	5.69	3.61	604.67			
Random effects						
Individual clusters of size 1	5.56	3.75	598.68	6.68		0.0110^a^
One large cluster	5.49	5.02	598.59	10.61		0.0174^a^
Artificial-clusters	5.59	3.95	599.29	6.63		0.0109^a^
Partially clustered						
Individual clusters of size 1	5.49	3.82	598.59	10.61		0.0174^b^
One large cluster	5.49	3.82	598.59	10.61		0.0174^b^
Artificial-clusters	5.49	3.82	598.59	10.61		0.0174^b^
Heteroskedastic						
Individual clusters of size 1	5.54	3.61	649.46	8.56	491.52	0.00744^b^
One large cluster	5.54	3.61	649.46	8.56	491.52	0.00744^b^
Artificial-clusters	5.54	3.61	649.46	8.56	491.52	0.00744^b^

^a^ICC across both arms of the trial

^b^ICC in the intervention arm only

**Table 6:** Summary of results for the Ulcer case study (*n*=233)

Model	Treatment estimate	Standard error	Residual variance	Random variance	Control variance	ICC
Ignore clustering	5.88	2.37	325.66			
Random effects						
Individual clusters of size 1	6.09	2.66	314.70	11.58		0.0355^a^
One large cluster	6.12	4.64	319.32	14.19		0.0425^a^
Artificial-clusters	6.08	2.92	315.18	11.96		0.0366^a^
Partially clustered						
Individual clusters of size 1	6.12	2.72	319.32	14.19		0.0425^b^
One large cluster	6.12	2.72	319.32	14.19		0.0425^b^
Artificial-clusters	6.12	2.72	319.32	14.19		0.0425^b^
Heteroskedastic						
Individual clusters of size 1	6.11	2.70	330.21	13.47	308.41	0.0207^b^
One large cluster	6.11	2.70	330.21	13.47	308.41	0.0207^b^
Artificial-clusters	6.11	2.70	330.21	13.47	308.41	0.0207^b^

^a^ICC across both arms of the trial

^b^ICC in the intervention arm only

**Table 7:** Summary of results for the PLINY case study (*n*=56)

Model	Treatment estimate	Standard error	Residual variance	Random variance	Control variance	ICC
Ignore clustering	6.83	5.35	398.13			
Random effects						
Individual clusters of size 1	6.83	5.35	398.13	<0.0001		<0.0001^a^
One large cluster	6.83	5.35	398.13	<0.0001		<0.0001^a^
Artificial-clusters	6.93	5.48	394.01	4.84		0.0121^a^
Partially clustered						
Individual clusters of size 1	6.83	5.35	398.13	<0.0001		<0.0001^b^
One large cluster	6.83	5.35	398.13	<0.0001		<0.0001^b^
Artificial-clusters	6.83	5.35	398.13	<0.0001		<0.0001^b^
Heteroskedastic						
Individual clusters of size 1	6.83	5.29	338.50	<0.0001	449.54	<0.0001^b^
One large cluster	6.83	5.29	338.50	<0.0001	449.54	<0.0001^b^
Artificial-clusters	6.83	5.29	338.50	<0.0001	449.54	<0.0001^b^

^a^ICC across both arms of the trial

^b^ICC in the intervention arm only

In the case studies considered, the estimates of the ICC are small with the largest value recorded for the Ulcer case study of 0.04 (Table 7) estimated using the partially clustered and random effects model (one large cluster). These small values may provide an explanation as to why the simple model ignoring clustering provides similar estimates to the more complex models in all four cases.

The results were replicated using Stata and the results were almost identical between the two packages.

## Discussion

In this paper, five different approaches to the analysis of iRCTs with clustering in the treatment arm have been discussed. Some of these approaches have been applied to four case studies in different settings to demonstrate their implementation and evaluate their use in practice.

The four case studies considered have small estimates for the ICC. All had an ICC less than 0.05 and three studies had an ICC less than 0.02. This indicates there was little clustering of outcomes. For example in the CPSW case studies the General Health Status score of a patient seen by Support Worker 1 would likely be similar had they been treated by Support Worker 2. As a result of the small cluster sizes and ICCs, we found little difference in the estimates of the treatment coefficients and their standard errors between four of the models. The ICCs observed in our case studies are not uncommon for trials of this nature. For example, in surgical trials with a quality of life endpoint (EQ-5D) identified by Cook et al. there were no trials with an ICC greater than 0.04 in either intervention arm [26]. Generally, we would expect the impact of the ICC to be larger when the therapist effect or cluster based intervention is delivered over a long period of time (so the ICC is high) or when the average size of the cluster is large (the ICC may be small in this case).

The simplest model, ignoring clustering, performed comparably well with the more complex models for all case studies. However, it is important to consider that this model does not truly reflect the design of the study as there was no allowance for clustering. We would not expect the simple model to perform well in circumstances where the ICC is higher or the cluster size is large. We do not recommend this model for use in practice, however, applying this model to our case studies illustrated that there was little difference in the results using the correct, more complex methods and so the results previously found are still valid.

Although in theory we might anticipate differences in the outcome of patients dependent on the cluster they belong to, in reality the ICC is often low as we observed in our case studies. One explanation could be, in clinical research practice, the training given to therapists as part of the protocol in some way standardises the treatment given. If the ICC was high there might be concern regarding the success of the intervention as there would be a strong reliance on the cluster and the therapist or healthcare professional delivering the intervention. We encourage the reporting of the ICC from clinical trials to aid the planning of future studies.

When using a random effects model to analyse clustering in just one arm of a study it is necessary to specify how the control arm is treated. In our results we found that for three of the case studies the choice of clustering for controls influenced the treatment coefficient estimates and their corresponding standard errors. Although this model performed well in our case, it does not truly represent the nature of clustering in the trial as we have forced clusters in the control arm that were not present in the actual trial. Therefore we do not recommend this approach.

The partially clustered and heteroskedastic models more accurately reflect the clustering in these trials, however, are of greater statistical complexity. We are often required to specify in advance of the trial commencing the proposed analysis. This is before any data from the trial has been collected so we do not know the ICC in the study. Therefore balancing the complexity of the model fitting procedure and the gain in accuracy of the results, we recommend to use the partially clustered model as a minimum, as this provides an accurate analysis of the study regardless of the observed ICC. We recommend, to allow fitting of the partially clustered models, participants in the unclustered arm are treated a clusters of size one as this provides a simple and intuitive solution for practical implementation.

If there is strong belief that there are different variances between the treatments and controls the heteroskedastic approach may be appropriate. We hope to identify, in a simulation study, a threshold value for the expected difference in individual level variability between the treatment and control arm whereby the heteroskedastic model will be more appropriate. Practically, fitting the partially clustered and heteroskedastic models in R and Stata required little additional work and the code to implement these models is provided.

### Limitations

This study employed a formal search of relevant literature to capture most of the related work conducted. However, this was not an exhaustive review of all work in this area. We have used four case studies that have arisen from our work as applied medical statisticians in clinical trials research. The results and inferences made are applicable to data with similar properties to these studies. For example our results focus only on continuous endpoints and as discussed relate to trials with small ICCs and relatively small clusters. All the case studies assumed each patient belonged to one cluster only; in the Acupuncture study patients only saw one therapist. We have not considered the effects of multiple membership [27].

Our analysis of these case studies was on complete cases only, we have ignored any data collected on patients for whom the outcome of interest was not recorded. The cluster allocation for participants in some of the case studies were also missing and we were not able to find this information. We therefore had to group these participants into one cluster. These data limitations may result in a large loss of information and potentially introduce bias, so alternative approaches should be explored. While small cluster sizes, small ICCs and incomplete data are issues in many real world data sets, to increase the generalisability of these results to trials with different characteristics to the case studies we hope to conduct a simulation study. This study will explore how the findings might change for varying cluster sizes, varying ICC, varying sample sizes and differential variance in the control and intervention arms.

We believe that while the control arm is ‘unclustered’, there is low level, natural clustering that occurs in practice in all trials - even trials with no formal clustering in either arm. For instance, patients in the unclustered control may be treated within the same hospital by healthcare professionals with similar skill levels or even by the same healthcare professional, creating potential dependencies in their outcomes. Baldwin et al. state that it is not plausible to have a non-zero ICC for the unclustered controls [5]. In their work they treat each individual in the cluster arm as their own cluster and conclude that the ICC for unclustered participants will have a negligible impact on estimation. Here we have considered clustering the controls as one large group cluster and using artificial clusters. We acknowledge that imposing these types of clusterings in the control arm that does not exist in reality could impact the estimation of the ICC. By imposing clustering on the control arm of the study we may impact the estimation of the ICC as we are either over or underestimating this low level natural clustering that is occurring. We will explore the impact of this imposed clustering on estimation in a simulation study.

In the analysis of cRCTs two popular methods of analysis include the use of random effects models and marginal models [2]. In this work we have chosen to use random effects models as this allowed the fitting of the more complex partially clustered and heteroskedastic models.

## Conclusions

In iRCTs where the treatment is group based or delivered by a health professional there is potential for a clustering of outcomes in the treatment arm only. As with any clustering this needs special attention in the design and analysis of the study. This paper has summarised the literature, identifying five potential approaches for the analysis of trials where there is clustering in one arm only. Some of these methods have been applied using the statistical packages R and Stata, exploring alternative methods to model the clustering in the control arm, to four case studies where clustering was present in one arm. Ignoring the clustering performed well for our case studies as a consequence of the low ICC in these studies. However, we do not recommend this approach in practice. Modelling homogeneous variances between the clustered and unclustered arm is adequate in scenarios similar to the case studies considered. We recommend treating each participant in the unclustered arm as a single cluster to facilitate modelling in a statistical package. This approach is simple to implement in R and Stata and is recommended for the analysis of trials with clustering in one arm only. The generalisability of our results is limited to trials similar to the case studies. Simulation work is required, for example, to determine scenarios where accounting for different levels of variability between treatment arms is necessary.

## Additional file

Additional file 1 Generic code. R and Stata code for models and ICC calculations. This file contains the R and Stata code required to implement the models used throughout this article. (DOCX 22 kb)

## Abbreviations

CPSW: Community Postnatal Support Worker
cRCT: Cluster randomised controlled trial
FE: Fixed Effects
GP: General Practitioner
Het: Heteroskedastic
ICC: Intra-cluster correlation coefficient
IQR: Inter-quartile range
iRCT: Individually randomised controlled trial
Min: Minimum
Max: Maximum
PC: Partially clustered
PLINY: Putting life in years
RCT: Randomised controlled trial
RE: Random effects
