# Supplementary material for: Recommendations for the analysis of individually randomised controlled trials with clustering in one arm – a case of continuous outcomes
Source: BMC Med Res Methodol. 2016 Nov 29;16:165. doi: 10.1186/s12874-016-0249-5 (PMC5129671; doi:10.1186/s12874-016-0249-5)
Supplement: Additional file 1 — Generic code. R and Stata code for models and ICC calculations. This file contains the R and Stata code required to implement the models used throughout this article. (DOCX 22 kb) [file 12874_2016_249_MOESM1_ESM.docx]

***Recommendations for the analysis of individually randomised controlled trials with clustering in one arm : A case of continuous outcomes***

**R and Stata code for models and ICC calculations**

# Variable Specification

The variables are defined as follows:

- outcome is the dependent variable;
- cluster is one of the following clustering options

1. cluster1 is the cluster indicator and treats the control group as clusters of size 1;
2. cluster2 is the cluster indicator and treats the control group as one cluster of size ;
3. cluster3 is the cluster indicator and treats the control group as random clusters of size ;

- treatment is a binary indicator for treatment (intervention group = 1, control group = 0).

# Generic R code

## Models

## Model 1: ignoring clustering

lm(outcome ~ treatment)

## Model 3: random effects

lmer(outcome ~ treatment + (1|cluster)) #NB: this requires the ‘**lme4**’ package

## Model 4: partial clustering

lmer(outcome ~ treatment + (0 + treatment|cluster)) #NB: this requires the ‘**lme4**’ package

## Model 5: heteroskedastic

lme(fixed=outcome ~ treatment,

random=~(0+treatment)|cluster,

weights=varIdent(form=~1|treatment),

na.action=na.omit,

method="REML") #NB: this requires the ‘**nlme**’ package

## ICC Calculations

## ICC for Models 3&4

ICCest(cluster, outcome) #NB: this requires the ‘ICC’ package

## ICC for Model 5

ICCFuncHet <- function(model){

random.var <- as.numeric(VarCorr(model)[1]) # between cluster variance (treatment)

control.var <- as.numeric(VarCorr(model)[2]) # between cluster variance (residual)

#http://r.789695.n4.nabble.com/How-to-extract-parameter-estimates-of-variance-function-from-lme-fit-td2997153.html (last accessed 15.12.15)

rsd.var <- (coef(model$modelStruct$varStruct, uncons = FALSE) * summary(model)$sigma) ^ 2 # residual variance

icc <- random.var / (random.var +control.var + rsd.var)

return(list(between_cluster.1=random.var,between_cluster.2=control.var, residual=rsd.var, icc=icc))

}

# Generic Stata Code

## Models

* Model 1: ignoring clustering

regress outcome treatment

* Model 3: random effects

mixed outcome treatment || cluster: , reml nolog

* Model 4: partial clustering

mixed outcome treatment || cluster: treatment , nocons reml nolog

* Model 5: heteroskedastic

mixed outcome treatment || cluster: treatment , nocons reml nolog residuals(independent, by(treatment))

#degrees of freedom correction for models 3 to 5 can be performed by including an option [, dfmethod(sat)] for Satterthwaitte correction or [.dfmethod(kr)] for Kenward-Roger correction

## ICC Calculations

*ICC for Models 3&4

estat icc

*ICC for Model 5

mat m=e(b)

local random_var=exp(m[1,3])^2

local rsd_var=exp(m[1,4])^2

local control_var=(exp(m[1,4]+m[1,5]))^2

di `random_var'/(`random_var'+`rsd_var'+`control_var') // displays the ICC
